# Selective, Temporary Postoperative Inhibition of Lymphangiogenesis by Integrin α5β1 Blockade Improves Allograft Survival in a Murine Model of High-Risk Corneal Transplantation

**DOI:** 10.3390/jcm13154418

**Published:** 2024-07-28

**Authors:** Tina Dietrich-Ntoukas, Felix Bock, Jasmine Onderka, Deniz Hos, Bjoern O. Bachmann, Grit Zahn, Claus Cursiefen

**Affiliations:** 1 Department of Ophthalmology, Charité—Universitätsmedizin Berlin, Corporate Member of Freie Universität Berlin and Humboldt Universität zu Berlin, 13353 Berlin, Germany; 2 Department of Ophthalmology, University Erlangen-Nürnberg, 91054 Erlangen, Germany; jasmine.onderka@uk-erlangen.de; 3 Department of Ophthalmology, University Hospital of Cologne, 50937 Cologne, Germany; felix.bock@uk-koeln.de (F.B.); deniz.hos@uk-koeln.de (D.H.); bjoern.bachmann@uk-koeln.de (B.O.B.); claus.cursiefen@uk-koeln.de (C.C.); 4 Eternygen GmbH, 10178 Berlin, Germany

**Keywords:** high-risk keratoplasty, allograft survival, lymhangiogenesis, CD11b+, integrin α5β1-blocking small molecules (JSM6427)

## Abstract

**Background:** Corneal inflammatory hem- and lymphangiogenesis significantly increase the risk for immune rejection after subsequent allogeneic corneal transplantation. The purpose of this study was to analyze the impact of temporary selective inhibition of lymphangiogenesis after transplantation on graft survival. **Methods:** Allogeneic transplantation from C57BL/6 mice to BalbC mice was performed as “high-risk” keratoplasty in a prevascularized corneal host bed (suture-induced inflammatory corneal neovascularization). The treatment group received integrin α5β1-blocking small molecules (JSM6427) at the time of transplantation and for two weeks afterwards. Control mice received a vehicle solution. Grafts were evaluated weekly for graft rejection using an opacity score. At the end of the follow-up, immunohistochemical staining of corneal wholemounts for lymphatic vessels as well as CD11b^+^ immune cells was performed. **Results:** Temporary postoperative inhibition of lymphangiogenesis by JSM6427 improved the corneal graft survival significantly. At the end of the follow-up, no significant reduction in CD11b^+^ immunoreactive cells within the graft compared to controls was found. **Conclusions:** The significant improvement of corneal graft survival by the selective, temporary postoperative inhibition of lymphangiogenesis after keratoplasty using integrin antagonists shows the impact of lymphatic vessels in the early postoperative phase. Retarding lymphatic vessel ingrowth into the graft might be sufficient for the shift to immunological tolerance in the postoperative period, even after high-risk keratoplasty.

## 1. Introduction

Corneal transplantation is experimentally widely used as a model for allogeneic transplantation [1,2]. As the cornea is a physiologically avascular tissue, corneal transplantation models can be used to analyze the impacts of lymphatic vessels (LVs) and blood vessels (BVs) on the graft outcome. The outgrowth of blood vessels and lymphatic vessels into the cornea (corneal hem- and lymphangiogenesis), before as well as after corneal transplantation, significantly increases the risk for immune rejection [3]. It has been shown for corneal allografts that LVs, pro-lymphangiogenic receptor VEGFR-3, and regional lymph nodes are essential for mediating immunological rejection [4,5,6,7,8,9].

The rate of immune reactions in avascular murine corneal graft beds is approximately 50% (representing the so-called normal- or low-risk transplantation: C57BL/6 to BALB/c), whereas the rate of immune reactions—by corneal suture placement—in prevascularized, so-called high-risk eyes reaches up to 100% [1,10,11]. The combined modulation of both hemangiogenesis and lymphangiogenesis after normal-risk corneal transplantation is able to improve graft survival in the murine model of corneal transplantation [8]. The removal of the regional lymph nodes, either after normal- or high-risk keratoplasty, led to nearly complete survival of the grafts in the sense of immune tolerance [6].

Pathological corneal lymphatic and blood vessels are a vector by which the so-called immune privilege of the physiologically avascular cornea is altered. The outgrowth of LVs is primarily mediated by the lymphatic growth factors VEGF-C and -D and their receptor VEGFR3 [12,13,14,15,16].VEGFR3 inhibition in the model of suture-induced inflammatory corneal neovascularization leads to the selective and almost complete inhibition of LV outgrowth without having a significant effect on hemangiogenesis [17]. Downstream signaling molecules such as tyrosine kinases and proteins such as integrins play an important functional role in lymphangiogenesis [18,19,20].

Integrins are ubiquitous heterodimeric proteins composed of α and β subunits and are important for cell–cell and cell–ECM connections [21,22]. They serve as transmembrane linkers between their extracellular ligands and the cytoskeleton and have the capacity to influence and regulate different processes such as cell migration during embryogenesis, angiogenesis, wound healing, immune and nonimmune defense mechanisms, hemostasis, and oncogenic transformation [21,23].

α5 integrins play an essential role during the development of the vascular system [24]. Integrin α5β1 and its extracellular ligand fibronectin are upregulated in tumor blood vessels and are involved in tumor angiogenesis and growth [25]. Integrins are also expressed on immune cells, e.g., α5ß1 is expressed on macrophages [26].

Integrin α5ß1 seems to be important for VEGFR-3-mediated cell signaling, because VEGFR-3 selectively associates with α5ß1, and tyrosine phosphorylation of VEGFR-3 can be blocked by an anti-α5ß1 antibody [27]. Integrin α5ß1 has been identified on lymphatic endothelial cells in vitro and in vivo by our group and others [18,27,28]. It was shown that the α5ß1 inhibitor JSM6427 [29] has an inhibitory effect on lymphatic endothelial cell proliferation in vitro as well as a dose-dependent differential effect on corneal hem- and lymphangiogenesis, allowing for the selective inhibition of lymphangiogenesis in vivo [18].

We already demonstrated the functional relevance of LVs for immune rejection after high-risk corneal transplantation: Selective anti-lymphangiogenic treatment by integrin α5ß1 inhibition or VEGFR-3 antibody prior to corneal grafting resulted in an improved graft survival in the presence of (hem-)vascularized and inflamed corneal beds. [7]. Compared to mice treated by VEGF-Trap_R1R2_ prior to transplantation—resulting in the inhibition of LVs, blood vessels, and reduced inflammation—the graft survival rate of mice under only anti-lymphangiogenic pre-operative treatment was equivalent [7].

The purpose of this study was to determine whether temporary selective inhibition of lymphangiogenesis using integrin α5ß1 antagonists immediately after high-risk allogeneic corneal transplantation (in a prevascularized corneal host bed) is able to improve intermediate-term graft survival by possibly interfering with sensitization and immune rejection. To address this question, we used the small-molecule integrin α5ß1 antagonist JSM6427 for two weeks after high-risk transplantation to specifically inhibit the outgrowth of LVs into the allograft. To our knowledge, this is the first study on selective anti-lymphangiogenic treatment using integrin antagonists after high-risk corneal transplantation.

## 2. Materials and Methods

### 2.1. Suture-Induced Inflammatory Corneal Neovascularization Assay

We used the mouse model of suture-induced inflammatory corneal neovascularization as described previously [10,11]. Six-week-old Balb/C mice (Charles River, Sulzfeld, Germany) were put under general anesthesia with an intramuscular injection of Ketanest^®^ S (8 mg/kg) and Rompun (0.1 mL/kg). Three intrastromal 11-0 nylon sutures (70-µm diameter needles; Serag-Wiesner, Naila, Germany) were placed in the cornea using a standardized method: The sutures were placed in the stroma with two incursions extending over 120° of corneal circumference each. The outer point of the sutures was chosen near the limbus, and the inner suture point was chosen near the corneal center at an equidistance from the limbus to induce standardized angiogenic responses. This procedure induces the combined outgrowth of blood vessels and LVs from the limbal arcade into the normally avascular cornea, resulting in an inflamed, hem-, and lymphvascularized host bed [30,31]. After 14 days, corneal sutures were removed. The animals were treated in accordance with the local animal care committee, the Association for Research in Vision and Ophthalmology Statement for the Use of Animals, and the “Principles of laboratory animal care”. The animal study protocol was approved by the Institutional Review Board of the University of Erlangen-Nürnberg, Germany (protocol codes 621.2531.31-13/04 and 621.2531.31-17/06). 

### 2.2. Allogeneic High-Risk Corneal Transplantation in Mice with a Vascularized Host Bed

Two weeks after the induction of inflammatory corneal neovascularization, so-called high-risk corneal transplantation (penetrating keratoplasty, PK) was performed as described in the following: 6-week-old Balb/C mice and C57BL/6 mice (Charles River, Sulzfeld, Germany) were put under general anesthesia with an intramuscular injection of Ketanest^®^ S (8 mg/kg) and Rompun (0.1 mL/kg). Donor corneas of the C57BL/6 mice were excised by trephination using a 2.0 mm bore and cut with curved Vannas scissors (Geuder, Germany). Corneal tissue was placed in chilled phosphate-buffered saline (PBS) until grafting. The graft beds of Balb/C mice were prepared by trephining a 1.5 mm site in the central cornea of the right eye and discarding the excised cornea. The donor cornea from a C57BL/6 mouse was immediately applied to the bed and sewed in with eight interrupted sutures (11-0 nylon, 70-µm diameter needles; Serag Wiessner, Naila, Germany). Afterwards, gentamicin ointment (MERCK PHARMA GMBH, Darmstadt, Germany) was placed on the corneal surface, and the eyelids were sutured with a 7-0 suture (Serag Wiessner, Naila, Germany) for tarsorrhaphy. Tarsal and corneal sutures were removed after 7 days, and grafts were then examined once a week until week 6 post-transplantation using slit lamp microscopy and scored for opacity, as described previously [32]. Clinical opacity scores of corneal grafts were as follows: 0, clear; +1, minimal, superficial (nonstromal) opacity, with the pupil margin and iris vessels readily visible through the cornea; +2, minimal, deep (stromal) opacity, with pupil margins and iris vessels visible; +3, moderate stromal opacity, with only the pupil margin visible; +4, intense stromal opacity, with only a portion of the pupil margin visible; and +5, maximum stromal opacity, with the anterior chamber not visible (shown in Supplemental Figure S1). Grafts with opacity scores higher than +2 after 2 weeks were considered to have been rejected, as has been described previously [32].

### 2.3. Normal-Risk versus High-Risk Model of Corneal Allogeneic Transplantation

Mice with allogeneic corneal transplantation in an avascular host bed (so called “low-risk” PK) served as a second control group. The procedure was performed as described above, but naive 6-week-old Balb/C mice without corneal neovascularization received the corneal allograft from C57/BL6 mice *(n* = 34). Intra- and postoperative care were the same as in high-risk PK (described above) as well as the postoperative graft evaluation.

### 2.4. Systemic Application of Small-Molecule Integrin Inhibitor JSM6427: Blocking Outgrowth of LVs during the Early Post-Transplantation Phase

In order to selectively inhibit the outgrowth of LVs, we used the inhibitory effect of the small-molecule integrin α5ß1 antagonist JSM6427 [18,28,29]. JSM6427 was provided by Jerini AG, Berlin, Germany.

Mice in the treatment group received JSM6427 systemically (23 mg/kg/d) via subcutaneous osmotic pumps (Alzet^®^ pumps, Durect Corp., Cupertino, CA, USA), as described previously [18], at the time of PK and for the following 14 days. This dose (23 mg/kg/d) has been shown to inhibit lymphangiogenesis selectively without having a significant effect on hemangiogenesis in the murine model of suture-induced corneal neovascularization [18]. Mice in the control group received the vehicle solution via osmotic pumps in the same way. The experiments were repeated 3 times, with 24 mice per group.

### 2.5. Immunohistochemical Analysis of Corneal Wholemounts at the End of the Follow-Up

After completion of the follow-up of 6 weeks, corneas were excised, rinsed in PBS, and fixed in acetone for 30 min, as described previously [30]. After three additional washing steps in PBS and blocking with 2% BSA in PBS for 2 h, the corneas were stained overnight at 4 °C with rabbit anti-mouse LYVE-1—a specific marker for lymphatic endothelium—(1:500; kind gift of D.G. Jackson, Oxford University, Oxford, UK), as described previously [8,30]. The following day, the tissue was washed, blocked, and stained with FITC-conjugated rat-anti-mouse CD11b (Serotec, Oxford, UK; diluted 1:100) antibody overnight at 4 °C. After a last washing and blocking step on day three, LYVE-1 was detected with a Cy3-conjugated secondary antibody (goat anti rabbit; 1:100; Dianova, Hamburg, Germany). Isotype control was performed with a FITC-conjugated normal rat2A IgG for CD31-FITC and CD11b and with a normal rabbit IgG (both Santa Cruz Biotechnology, Santa Cruz, CA, USA) for LYVE-1.

Fluorescence microscopy and photography were performed using the Olympus BX51 fluorescence microscope (Olympus Optical Co., Hamburg, Germany) and the F-View II monochrome CCD camera (Soft Imaging System, Münster, Germany), with software provided by analySIS^B (Soft Imaging Systems, Münster, Germany). Each wholemount image was created by assembling nine digital images taken at 100× magnification (analySIS^B; Soft Imaging System, Münster, Germany).

### 2.6. Functional and Statistical Analyses

Postoperative corneal allograft survival was analyzed using Kaplan–Meier survival curves. Quantitative analysis of LVs was performed in a standardized procedure using analySIS^B (Soft Imaging System, Münster, Germany) software by means of threshold analysis. To improve the objectivity and precision of the morphometrical analysis, we established a modified method using grey filter sampling on monochromatic pictures, which we described previously [33]: Grey-scale images of the wholemount photographs were modified using several filters and then analyzed using a standardized algorithm established in an image analysis program (analySIS^B; Soft imaging system; Münster, Germany), which is shown in Supplemental Figure S2). Lymphatic vessels and inflammatory cells were detected with a threshold setting that included the bright structures (LYVE-1-positive lymphatic vessels, CD11b-positive immunoreactive cells) and excluded the dark background. The corneal area filled with LVs (lymphvascularized area) was measured. The results of the control group were defined as being 100%. To determine the amount of CD11b-positive immunoreactive cells in the cornea, a standardized rectangle in the central cornea was analyzed using threshold analysis, as described above. The area filled with CD11b-positive cells was measured. Statistical analysis was conducted using InStat 3 Version 3.06 (GraphPad Software Inc., San Diego, CA, USA). Graphs were drawn using Prism4, Version 4.03 (GraphPad Software Inc, San Diego, CA, USA).

## 3. Results

### 3.1. Improvement of Corneal Graft Survival by Treatment with JSM6427, a Specific Small-Molecule Antagonist against Integrin α5ß1, after Allogeneic High-Risk Keratoplasty

**Figure 1:**
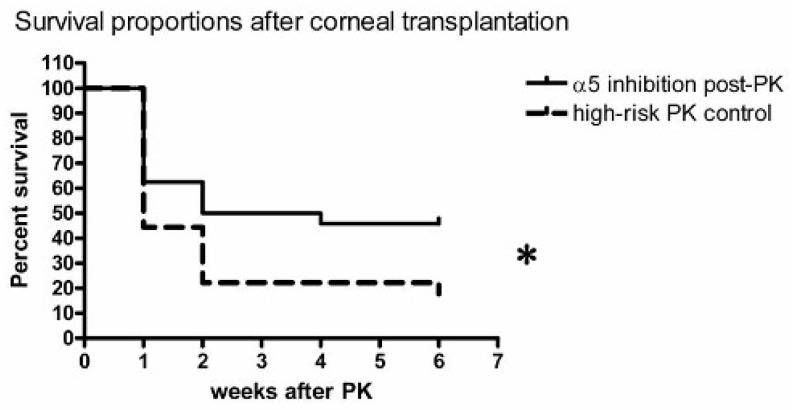
Systemic integrin α5β1 inhibition for 14 days after high-risk corneal transplantation (PK) (*n* = 23) significantly (*) improved corneal graft survival compared to control (*n* = 22) high-risk penetrating keratoplasty (*p* = 0.021) and reduced the risk of rejection to the level of low-risk PK. Mice treated with anti-lymphangiogenic JSM6427 after high-risk transplantation in a prevascularized host bed (treatment starting at day of surgery, continued for 14 days) showed the same level of graft survival after high-risk PK (vascularized corneal host beds) as normal-risk corneal transplantations (*n* = 34) in non-vascularized corneal host beds (Kaplan–Meier survival curves; shown in Supplemental Figure S3).

Systemic integrin α5β1 inhibition by small molecules (JSM6427) for 14 days after high-risk corneal transplantation significantly improved corneal graft survival compared to control “high-risk” PK (*p* = 0.021) and reduced the risk of rejection to the level of low-risk PK: Mice treated with anti-lymphangiogenic JSM6427 after transplantation (treatment starting at the day of surgery and continued for 14 days) showed the same level of graft survival after high-risk PK as the second control group of normal-risk corneal transplantation in non-vascularized corneal host beds (Kaplan–Meier survival curves; Figure 1, Supplemental Figure S3). The positive effect on graft survival is comparable to the effect seen with JSM6427 treatment for 2 weeks before corneal grafting that we described previously [7]. We saw no signs of deteriorated wound healing and no higher rate of suture loosening in the treatment group. In the final analysis were 22 (control group) and 23 (treatment group) mice per group, as two mice in the control group and one mouse in the treatment group had to be excluded.

### 3.2. Intermediate-Term Effect of Integrin α5ß1 Blockade on Lymphatic Vessels in the Graft

**Figure 2:**
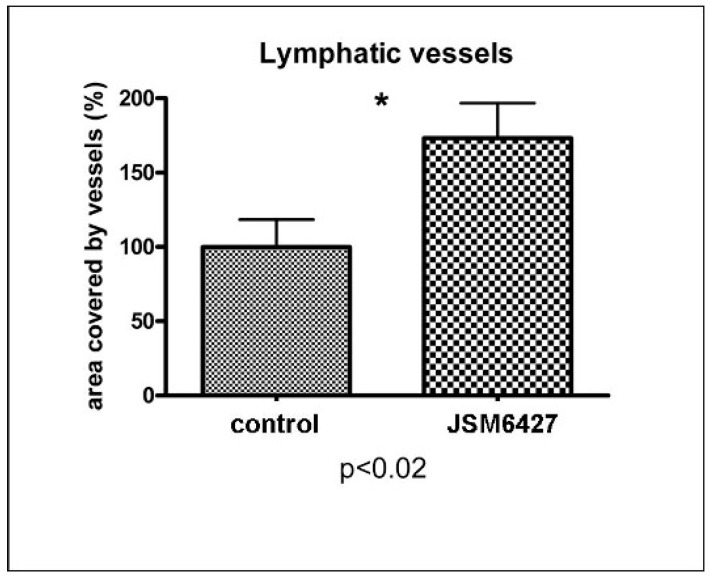
Corneal grafts displayed significantly (*) more LVs after 6 weeks (end of follow-up) compared to control high-risk keratoplasties (*p* < 0.02; lymphvascularized area % vs. control set as 100%) when mice were treated by the integrin α5ß1 inhibitor JSM6427 for two weeks immediately after high-risk corneal transplantation in a prevascularized host bed. The temporary, postoperative anti-lymphangiogenic treatment, therefore, does not seem to have a prolonged effect. However, retarding lymphatic vessel growth early after transplantation seems to be sufficient for an improved intermediate-term graft survival in high-risk keratoplasty despite the later existence of LVs in the graft.

While the systemic inhibition of integrin α5ß1 by JSM6427 for 14 days prior to corneal transplantation [7] resulted in a statistically significant reduction of LVs in the graft even 6 weeks after high-risk PK compared to the control group (*p* = 0.0023; lymph-vascularized area % vs. control set as 100%; [7], temporary treatment with JSM6427 for two weeks after transplantation had no prolonged inhibitory effect on lymphangiogenesis. In contrast, there were even more LVs in the graft at the end of the follow-up (after 6 weeks) (*p* < 0.02; lymphvascularized area % vs. control set as 100%; Figure 2).

### 3.3. Intermediate-Term Effect of Integrin α5ß1 Blockade by JSM6427 on the Amount of CD11b+ Cells in the Grafted Cornea

**Figure 3:**
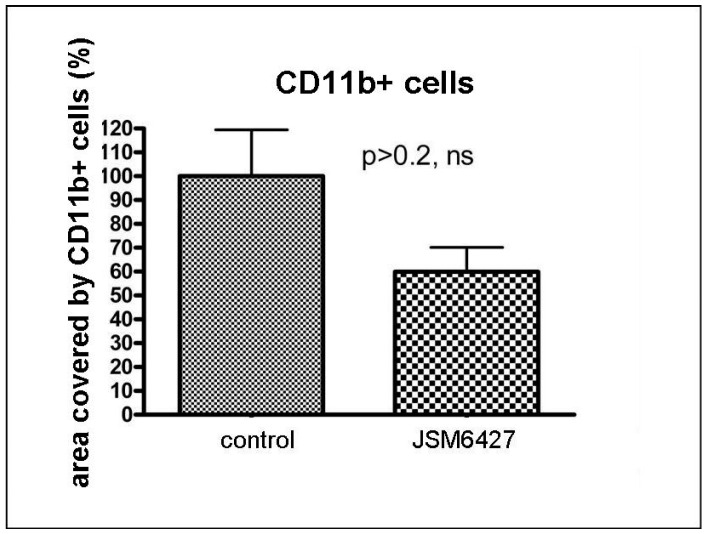
The number of CD 11b-positive immunoreactive cells in the corneal graft at the end of the follow-up period (6 weeks after transplantation) was not significantly reduced when mice were treated by the integrin α5ß1 inhibitor JSM6427 for two weeks immediately after transplantation (*p* > 0.02, n.s.; control set as 100%).

The treatment with the integrin α5ß1 inhibitor JSM6427 had no prolonged effect on macrophage recruitment into the grafted cornea after high-risk corneal transplantation: Integrin α5ß1 blockade for 2 weeks after transplantation reduced the number of CD11b-positive cells in the graft at the end of the follow-up period (6 weeks) compared to the control, but this effect was not significant (*p* > 0.02, n.s, Figure 3).

## 4. Discussion

By the specific blockade of single functional steps of the angiogenic cascade, it is possible to not only inhibit angiogenesis in total but to also selectively inhibit LV outgrowth [17,18]. This possibility of molecular discrimination of lymph- and hemangiogenesis led to the concept of analyzing the specific impact of LVs on the risk of graft rejection after corneal transplantation.

Previously, we showed that selective inhibition of lymphangiogenesis prior to corneal transplantation in the high-risk setting promotes graft survival [7]. Since, in real-life settings, patients often present with already established corneal neovascularization, the question is whether selective postoperative inhibition of additional lymphangiogenesis is able to promote graft survival. In the present study, we show that the selective postoperative inhibition of lymphatic vessel ingrowth has a positive effect on graft survival after high-risk keratoplasty in a prevascularized host bed.

Potential clinical strategies of anti-lymphangiogenic treatment that reduce the risk of graft rejection after corneal transplantation may be as follows: 1. the inhibition of LV outgrowth into the grafted cornea (grafting itself being an angiogenic stimulus), 2. The regression of pre-existing lymphatic vessels in the vascularized host beds, and 3. interference with the recruitment of antigen-presenting cells (such as macrophages) into corneal LVs [31]. The benefit of a selective blockade of only lymphangiogenesis may be better for wound healing, especially if this novel concept will be transferred to extraocular transplant settings such as other tissue or organ transplants [34,35].

We have shown that corneal allografts placed in only hem- and not lymphvascularized host beds display better survival rates [7]. Here, we demonstrate that the temporary postoperative specific blockade of lymphangiogenesis via the inhibition of integrin α5ß1 *after* transplantation is also able to increase graft survival significantly. Survival rates improve up to the level of normal-risk survival rates, as achieved with the anti-hemangiogenic and anti-lymphangiogenic treatments with VEGF-Trap_R1R2_ [8].

Apparently, a temporary, postoperative inhibition of (additional) lymphangiogenesis seems to be sufficient for improved intermediate-term graft survival in high-risk keratoplasties despite the later existence of LVs in the graft. Retarding lymphangiogenesis after transplantation may narrow the time of opportunity during which recipient sensitization takes place and may, therefore, promote a shift in the balance of the recipient alloimmune response toward immunological tolerance.

The concept of postoperative antiangiogenesis in acute [36] and chronic regressed [37] high-risk eyes is not new. Our novel finding here is the surprising effect of selective lymphangiogenesis inhibition postoperatively.

The finding that the specific inhibition of lymphangiogenesis contributes to a better graft survival after high-risk PK does not implicate that LVs are the only route for the generation of an immune response: Blocking VEGFR3 leads to a decreased rate of graft-derived APCs in the draining lymph nodes of the eye in the absence of LVs; after 72 h in both the VEGFR3 antibody-treated and the control group, no LVs reached the host–graft border, even though APCs were detected in the draining lymph nodes after 48 h [9]. VEGFR3 seems to mediate corneal dendritic cell migration to the lymph and, thereby, the induction of immunity to the corneal transplant in the absence of LVs [9].

Integrins and their receptors are responsible for complex cellular functions and interactions; therefore, mechanisms other than the inhibition of lymphangiogenesis may contribute to the improved graft survival in JSM6427-treated mice. Macrophage interactions may also be altered by integrin α5ß1 antagonists, because integrin α5ß1 is expressed on macrophages and dendritic cells [26]. However, the blockade of integrin α5ß1 by JSM6427 did not significantly impair the amount of CD11b+ immunoreactive cells into the cornea in the suture-induced model of corneal inflammatory neovascularization [18]. This primarily unexpected effect has also been found in a model of airway inflammation [28]. It may be due to the fact that macrophages are recruited, amongst others, by VEGF-A via VEGFR1, not just by VEGF-C via VEGFR3 [30]. At the end of the follow-up, we did not find significantly reduced CD11b-positive cells in the corneal graft in this model of corneal transplantation. Nevertheless, the functioning of these immunoreactive cells may be altered by the treatment: It has been shown recently that blocking the activated leukocyte cell adhesion molecule (ALCAM, CD166) leads to the retention of dendritic cells in corneas and effectively prevents corneal allograft rejection [38].

The existence of LVs after 6 weeks in corneal grafts of mice despite anti-lymphangiogenic treatment for 2 weeks immediately after PK might be a rebound effect: Retarding LV ingrowth into the graft might be sufficient for the shift to immunological acceptance in the postoperative period (e.g., because regulatory T-cells emerge after 2 to 4 weeks), while the later presence of LVs may contribute to the clearance and homeostasis of the graft without triggering allograft rejection. Our findings contribute to the concept that the role of LVs in allografts may go beyond sensitization: They seem to be involved in mechanisms of immune tolerance, homeostasis, and clearance of the graft. Their role for the immunological host response to the graft (rejection vs. tolerance) and homoeostasis after solid organ transplantation is complex and has to be further explored in the future [34,39,40,41,42,43]. In a model of experimental lung transplantation, acute graft rejection was associated with the cessation of lymphatic drainage from the graft [44]. LV density also correlates with better renal graft function at one year after kidney transplantation [42]. A significantly lower density of VEGFR-3-positive LVs was observed in patients with severe graft rejections after heart transplantation [39]. In contrast, Yamamoto and colleagues found that LVs are strongly associated with inflammatory processes in the graft after kidney transplantation [43]. In addition, Kerjaschki and colleagues proposed that newly formed LVs organize perivascular lymphocytes into immunologically active follicular structures [34,40,41]. Because solid organ grafts—unlike the physiologically avascular cornea—have the need for blood supply in order to achieve graft survival, specific (temporary) anti-lymphangiogenic therapies may be of great value for future transplantation strategies.

## 5. Conclusions

We have shown that the temporary selective postoperative inhibition of (additional) lymphangiogenesis by an integrin α5β1 blockade shortly after high-risk transplantation of corneal allografts reduces the risk of immunological graft rejection. Selective temporary inhibition of lymphangiogenesis, therefore, may be a novel tool to improve allograft survival without significantly affecting blood supply or wound healing.

## Data Availability

The raw data supporting the conclusions of this article will be made available by the authors on request.

## Supplementary Materials

The following supporting information can be downloaded at: https://www.mdpi.com/article/10.3390/jcm13154418/s1: Figure 1: Examples for clinical scores of corneal grafts for opacity: (a) +1: minimal, superficial (nonstromal) opacity; pupil margin and iris vessels readily visible through the cornea; (b) +2: minimal, deep (stromal) opacity; pupil margins and iris vessels visible; (c) +3: moderate stromal opacity; only pupil margin visible; (d) +4: intense stromal opacity; only a portion of pupil margin visible. Figure 2: Semiautomatic quantification of corneal lymphangiogenesis: (a) original image of a corneal flat mount stained with Lyve-1; (b) detected and quantified vessels (red) as described in reference [33]. Figure 3: Systemic integrin α5β1 inhibition for 14 days after high-risk corneal transplantation (PK) (*n* = 23) significantly improved corneal graft survival compared to control (*n* = 22) high-risk penetrating keratoplasty (*p* = 0.021;) and reduced the risk of rejection to the level of low-risk PK. Mice treated with anti-lymphangiogenic JSM6427 after high-risk transplantation in a prevascularized host bed (treatment starting at day of surgery, continued for 14 days) showed the same level of graft survival after high-risk PK (vascularized corneal host beds) as normal-risk corneal transplantations (*n* = 34) in non-vascularized corneal host beds (Kaplan-Meier survival curves).
