# Supplementary material for: Selective, Temporary Postoperative Inhibition of Lymphangiogenesis by Integrin α5β1 Blockade Improves Allograft Survival in a Murine Model of High-Risk Corneal Transplantation
Source: J Clin Med. 2024 Jul 28;13(15):4418. doi: 10.3390/jcm13154418 (PMC11313630; doi:10.3390/jcm13154418)
Supplement: Supplementary file 1 [file jcm-13-04418-s001.zip › Suppl Figures_legends.pdf]

Supplementary Figure S1: Examples for clinical scores of corneal grafts for opacity: a) +1: minimal, superficial (nonstromal) opacity; pupil margin and iris vessels readily visible through the cornea; b) +2: minimal, deep (stromal) opacity; pupil margins and iris vessels visible; c) +3: moderate stromal opacity; only pupil margin visible; d) +4: intense stromal opacity; only a portion of pupil margin visible.

Supplementary Figure S2: Semiautomatic quantification of corneal lymphangiogenesis: a) original image of a corneal flat mount stained with Lyve-1; b) detected and quantified vessels (red) as described in: Bock F, Onderka J, Hos D, Horn F, Martus P, Cursiefen C. Improved semiautomatic method for morphometry of angiogenesis and lymphangiogenesis in corneal flatmounts. Exp Eye Res. 2008 Nov;87(5):462-70. doi: 10.1016/j.exer.2008.08.007. Epub 2008 Aug 26. PMID: 18789928.

Supplementary Figure S3: Systemic integrin $\alpha5\beta1$ inhibition for 14 days *after* high-risk corneal transplantation (PK) (n=23) significantly improved corneal graft survival compared to control (n=22) high-risk penetrating keratoplasty (p = 0.021;) and reduced the risk of rejection to the level of low-risk PK. Mice treated with anti-lymphangiogenic JSM6427 after high-risk transplantation in a prevascularized host bed (treatment starting at day of surgery, continued for 14 days) showed the same level of graft survival after high-risk PK (vascularized corneal host beds) as normal-risk corneal transplantations (n=34) in non-vascularized corneal host beds (Kaplan-Meier survival curves).
