# Supplementary figures and images for: Selective, Temporary Postoperative Inhibition of Lymphangiogenesis by Integrin α5β1 Blockade Improves Allograft Survival in a Murine Model of High-Risk Corneal Transplantation
Source: J Clin Med. 2024 Jul 28;13(15):4418. doi: 10.3390/jcm13154418 (PMC11313630; doi:10.3390/jcm13154418)

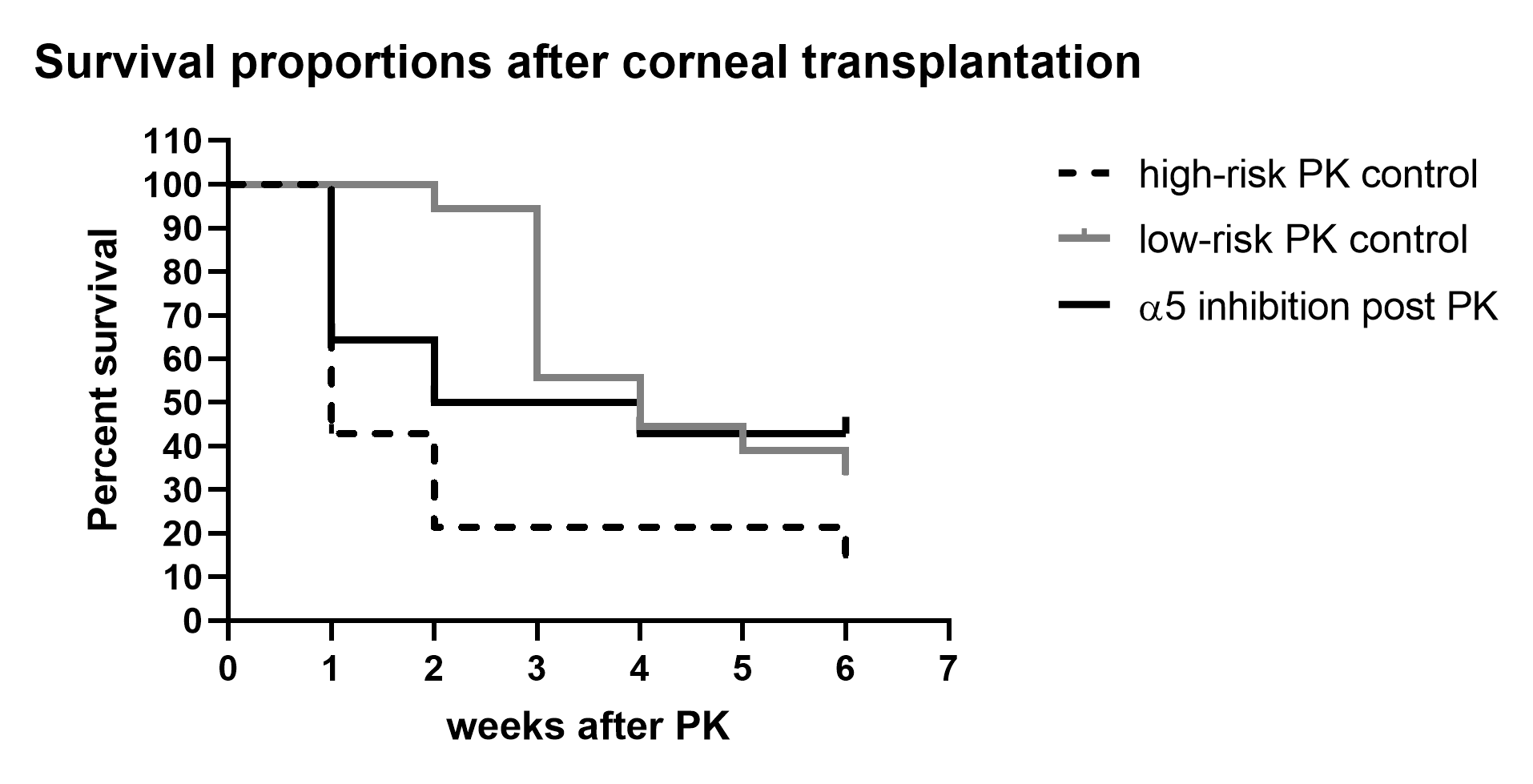

Supplement: Supplementary file 1 [file jcm-13-04418-s001.zip › Suppl Figure 3.tiff]
